# High LIN28A Expressing Ovarian Cancer Cells Secrete Exosomes That Induce Invasion and Migration in HEK293 Cells

**DOI:** 10.1155/2015/701390

**Published:** 2015-10-25

**Authors:** Vanessa A. Enriquez, Ellane R. Cleys, Juliano C. Da Silveira, Monique A. Spillman, Quinton A. Winger, Gerrit J. Bouma

**Affiliations:** ^1^Cell and Molecular Biology Program, Department of Biomedical Sciences, Colorado State University, Fort Collins, CO 80523-1683, USA; ^2^Animal Reproduction and Biotechnology Laboratory, Department of Biomedical Sciences, Colorado State University, Fort Collins, CO 80523-1683, USA; ^3^Department of Veterinary Medicine, Faculty of Animal Science and Food Engineering, University of São Paulo, 13635-900 São Paulo, SP, Brazil; ^4^Department of Obstetrics and Gynecology, University of Colorado Denver School of Medicine, Aurora, CO 80045, USA

## Abstract

Epithelial ovarian cancer is the most aggressive and deadly form of ovarian cancer and is the most lethal gynecological malignancy worldwide; therefore, efforts to elucidate the molecular factors that lead to epithelial ovarian cancer are essential to better understand this disease. Recent studies reveal that tumor cells release cell-secreted vesicles called exosomes and these exosomes can transfer RNAs and miRNAs to distant sites, leading to cell transformation and tumor development. The RNA-binding protein LIN28 is a known marker of stem cells and when expressed in cancer, it is associated with poor tumor outcome. We hypothesized that high LIN28 expressing ovarian cancer cells secrete exosomes that can be taken up by nontumor cells and cause changes in gene expression and cell behavior associated with tumor development. IGROV1 cells were found to contain high LIN28A and secrete exosomes that were taken up by HEK293 cells. Moreover, exposure to these IGROV1 secreted exosomes led to significant increases in genes involved in Epithelial-to-Mesenchymal Transition (EMT), induced HEK293 cell invasion and migration. These changes were not observed with exosomes secreted by OV420 cells, which contain no detectable amounts of LIN28A or LIN28B. No evidence was found of LIN28A transfer from IGROV1 exosomes to HEK293 cells.

## 1. Introduction

Epithelial ovarian cancer (EOC) is the most lethal gynecological malignancy worldwide and is often detected in late stages where metastasis has occurred [1]. In ovarian cancer, tumor cells release small cell-secreted vesicles called exosomes [2–4]. Exosomes are endosome-derived vesicles (30–100 nm) that contain bioactive materials and are released by cells into the bloodstream [5], as well as urine [6], saliva [7] plasma [8], epididymal fluid [9], amniotic fluid [10], follicular fluid [11], malignant and pleural effusions of ascites [12], bronchoalveolar lavage fluid [13], synovial fluid [14], and breast milk [15]. Exosomes are also known to affect gene expression as Valadi and colleagues demonstrated RNAs in mast cell exosomes could be delivered to human and mouse mast cells leading to new protein production in recipient cells [2]. Moreover, tumor cell-secreted exosomes can induce increased cell proliferation and invasion in target cells [4, 16–18].

MicroRNAs (miRNAs) are abundantly expressed in human cancers [19, 20]. There are unique miRNA signatures representative of human cancers [21], including ovarian cancer [22] implying miRNAs are key regulators of cellular and molecular function contributing to metastatic disease. miRNAs are nonprotein coding RNAs that function as posttranslational regulators by binding to the 3′UTR of target mRNAs [23]. They are evolutionarily conserved and approximately 19–22 nucleotides in length. Upon binding to the 3′UTRs of target mRNAs, translational inhibition occurs in the form of mRNA target cleavage or translational repression [24]. The dynamic roles miRNAs have on mRNA target genes can alter signaling pathways associated with the hallmarks of cancer [25]. Furthermore, miRNAs also are present in exosomes and can be delivered from one cell to another [2].

LIN28 is a RNA-binding protein that regulates both mRNA and miRNAs. There are two paralogs of LIN28, LIN28A, and LIN28B, both containing a cold shock domain (CSD) and CCHC-zinc finger RNA-binding domain. They regulate* let-7* miRNA levels by CSD binding to the NGNGAYNNN (N = any base and Y = pyrimidine) sequence on the terminal loop of* let-7* and CCHC-zinc finger binding to the GGAG sequence on the same terminal loop [26]. The linker between the CSD and the CCHC-zinc finger allows for binding of all twelve* let-7* miRNA family members. Studies have focused on elucidating the role of LIN28 and* let-7s* miRNAs in cancer cells [27]; high LIN28A levels are associated with advanced human malignancies [28] and LIN28A is often expressed in ovarian tumors [29, 30]. Considering the positive correlation between LIN28 level and tumor aggressiveness, as well as the observation that tumors are known to secrete exosomes that can induce proliferation, invasion, and/or migration, it is possible that high LIN28 level in cells positively regulates secretion of exosomes with oncogenic potential.

The goal of this study was to test the hypothesis that exosomes from ovarian cancer cells that contain high LIN28 can be taken up by HEK293 cells and lead to changes in gene expression and cell phenotype, whereas exosomes from ovarian cancer cells with low LIN28 levels cannot. To this end we used IGROV1 and OV420 cells; IGROV1 cells can induce peritoneal carcinomatosis in SCID mice, leading to rapid tumor formation and cell growth [31], while OV420 cells do not form tumors in SCID mice [32]. (Figure S1 in Supplementary Material available online at http://dx.doi.org/10.1155/2015/701390).

## 2. Material and Methods

### 2.1. Cell Lines and Culture Conditions

IGROV1 and OV420 cell lines were cultured in Roswell Park Memorial Institute (RPMI 1640) medium with L-glutamine 1X (Cellgro, 10-040-CV), supplemented with 10% fetal bovine serum (FBS) (Atlas Biologicals, F-0500-D) and 1% antibiotic-antimycotic solution (Cellgro, 30-0004-Cl). HEK293 (human embryonic kidney) cells were kindly provided by Dr. Russell Anthony (Colorado State University) and were cultured in Dulbecco's Modified Eagle Medium (DMEM) (Cellgro, 10-017-CV) supplemented with 10% fetal bovine serum (Atlas Biologicals, F-0500-D) and 1% antibiotic-antimycotic solution (Cellgro, 30-0004-Cl). Cells were cultured in a standard humidified incubator at 37°C in a 5% CO_2_ atmosphere.

### 2.2. Lentiviral Transductions for Exosome Tracking

IGROV1 cells line were stably transduced with pCT-CD63-GFP Cyto-tracers (System Biosciences, CYTO120-VA-1) to create an IGROV1-CD63-GFP cell line used for exosome tracking as per manufacturer's instructions. Briefly, 1 × 10^3^ IGROV1 cells were seeded onto 24-well plates 24 hours before transfection to allow adhesion and were grown to approximately 60–80% confluency. The cells were transduced with pCT-CD63-GFP at a multiplicity of infection (MOI) of 2000 virus particles per cell with addition of Polybrene (Millipore, TR-1003-G) at a final concentration of 2 *μ*g/mL to increase efficiency of transfection. Cells were incubated for 24 hours and 1-day later fresh medium was added without puromycin for another 24 hours. Infected IGROV1 cells were selected by adding puromycin at a final concentration of 4 *μ*g/mL.

### 2.3. Exosome Isolation

Complete RPMI 1640 and DMEM medium was ultracentrifuged (Beckman L8-80) at 100,000 g for 16 hours at 4°C to pellet secreted membrane vesicles less than 1000 nm to obtain vesicle-depleted medium. Sterile filtration was performed on vesicle-depleted medium using a 0.2 *μ*m PES membrane (Thermo Scientific, 565-0020) and stored at 4°C until exosome collection.

For exosome isolation, 1 × 10^6^ cell were seeded onto four 10 cm cell plates (Celltreat, 229690) and cultured in either RPMI 1640 vesicle-depleted medium or DMEM vesicle-depleted medium for three days. Supernatant was collected and centrifuged at 3,000 g for 15 minutes at 4°C to remove cells and cell debris. Supernatant and ExoQuick-TC Exosome precipitation solution (System Biosciences, EXOTC50A-1) were combined in a 5 : 1 dilution (resp.) and exosomes were collected as per manufacturer's instructions. Briefly, supernatant/ExoQuick-TC biofluid was centrifuged at 1,500 g for 30 minutes at 4°C; biofluid was aspirated and recentrifuged at 1,500 g for 5 minutes at 4°C to remove excess biofluid without disturbance of exosome pellet. Four exosome pellets were combined and either resuspended in 200 *μ*L of TRIzol LS Reagent (Life Technologies, 10296028) for qPCR or 300 *μ*L of M-PER (Thermo Scientific, 78501) supplemented with Halt proteinase inhibitor cocktail (Thermo Scientific, 1 : 100, 87786) and phenylmethanesulfonyl fluoride solution (Boston BioProducts, 1 : 100, PI-120). Exosomes were stored at −80°C until RNA or protein was isolated.

### 2.4. RNA Isolation

Total RNA was extracted from confluent cells lysed in 300 *μ*L of MirVana lysis binding buffer and 30 *μ*L of miRNA homogenate additive. RNA was isolated per manufacturer's instructions using MirVana miRNA isolation kit (Ambion, AM1561) and resuspended in 30 *μ*L of RNase/DNase-free water.

Total RNA was isolated from exosome isolates using TRIzol LS Reagent (Life Technologies, 10296-028). RNA isolation was completed per manufacturer's instructions with minor modifications. Briefly, exosomes were lysed in 200 *μ*L of TRIzol LS Reagent (Life Technologies, 10296-028) and homogenized for 5 minutes. Phase separation was conducted by adding 128 *μ*L of chloroform to the RNA/DNA/protein phase and homogenization for 5 minutes. Samples were centrifuged for 15 minutes at 4°C to separate the RNA, DNA, and protein phases. The RNA aqueous phase was added to 400 *μ*L of cold 100% isopropanol and stored at −80°C overnight for RNA precipitation. RNA was pelleted via centrifugation and was washed twice with cold 75% ethanol and then resuspended in 10 *μ*L of RNase/DNase-free water.

Once total RNA was isolated from both cells and exosomes, DNase-free DNase Treatment and Removal kit (Ambion, AM1906) was used on all samples to eliminate genomic DNA contamination. RNA quality and concentration were assessed using the NanoDrop ND-1000 spectrophotometer (NanoDrop Technologies, USA). Total RNA absorbance of 260/280 was measured and samples with RNA purity between 1.7 and 2.2 were used for experiments. Samples were stored at −80°C until qPCR was performed.

### 2.5. Reverse Transcriptase PCR (RT-PCR)

RT-PCR was performed from total RNA (see above) where 1 *μ*g of RNA was used for cells and 400 ng of RNA was used for exosomes utilizing qScript cDNA Supermix (Quanta Biosciences, 95047-100) as per manufacturer's instructions. Once cDNA was made, the GoTaq DNA Polymerase kit (Promega, M3005) was used with either LIN28A or LIN28B primers. PCR cycling parameters were an initial denaturation step for 5 minutes at 94°C followed by 40 cycles of 15 seconds at 94°C of denaturation, 30 seconds at 60°C for annealing and 15 seconds at 72°C for elongation, and a final elongation step of 3 minutes at 72°C. Amplicons were electrophoresed on a 2% agarose gel at 190 V for 30 minutes and imaged using the ChemiDoc MP System with the Image Lab 4.1 software. Experiments were carried out using three independent biological replicates and the experiments were repeated. The primers used were designed to span introns to ensure no DNA contamination was present. LIN28A forward primer sequence was 5′-GGCATCTGTAAGTGGTTGAACG-3′ and the reverse primer sequence was 5′-CCTTCCATGTGCAGCTTACTCT-3′ (118 bp size) and LIN28B forward primer sequence was 5′-TAGGAAGTGAAAGAAGACCCAA-3′ and the reverse primer sequence was 5′-ATGATGCTCTGACAGTAATGG-3′ (151 bp size).

### 2.6. Quantitative Real-Time PCR (qPCR)

Taqman qPCR was performed on cDNA generated from total RNA (see above). cDNA was diluted to a final concentration of 10 ng/rxn for cells and 20 ng/rxn for exosomes and combined with 2x Ssofast Probe Supermix (Bio-Rad, 172-5230) and 20x Taqman Assay Mix (Applied Biosystems). The 20x Taqman Assay Mix Probes (Applied Biosystems) used for this study were as follows:* LIN28A* (Hs00702808_s1),* LIN28B* (Hs01013729_m1),* GAPDH* (H99999905_m1),* MRPS15* (Hs00229834_m1), and* TBP* (Hs00427620_m1). qPCR was performed using the LightCycler 480 Real-Time PCR System (Roche Applied Science). PCR cycling parameters were an initial denaturation step for 30 seconds at 95°C followed by 45 cycles of repeating denaturing at 15 seconds at 95°C and annealing for 30 seconds at 60°C with a final cooling cycle for 5 minutes at 37°C. Data were normalized using the geometric mean of* GAPDH*,* MRPS15*, and* TBP* and relative levels were calculated using the comparative Cp method to obtain 2^−ΔCt^ relative expression values. Each sample was run in duplicate with reverse transcriptase negative controls, nontemplate controls, and experiments were repeated. Statistical analysis was determined by analysis of variance (ANOVA) followed by Tukey pairwise comparison (Minitab 17). *p* values less than 0.05 were considered statistically significant.

miRNA qPCR was performed using cDNA diluted to a final concentration of 1.5 ng/rxn and combined with 2x QuantiTect SYBR green PCR master mix, 10x miScript Universal Primer (miScript SYBR Green PCR Kit, Qiagen, 218075). The miRNA PCR primers used for this study are listed in Table 1. qPCR was performed using the LightCycler 480 Real-Time PCR System (Roche Applied Science). The cycling conditions were an initial denaturation step for 15 minutes at 95°C followed by 45 cycles of repeating denaturing at 15 seconds at 95°C, annealing for 30 seconds at 55°C, elongation for 30 seconds at 72°C. Melt curves were generated to ensure single miRNA amplicons using the following cycling parameters: 95°C for 5 seconds and 65°C for 1 minute. Data were normalized using snRNA (U6), and relative levels were calculated using the comparative Cp method. Each sample was run in duplicate with reverse transcriptase negative controls, nontemplate controls, and experiments were repeated. Statistical analysis was determined by ANOVA followed by Tukey pairwise comparison (Minitab 17). *p* values less than 0.05 were considered statistically significant. A two-sided unpaired Student's *t*-test was performed on miRNA levels after exosome transfer, with significance at *p* < 0.05.

Human Epithelial-to-Mesenchymal Transition (EMT) RT^2^ Profiler PCR array (SABiosciences, PAHS-090G-4) was used to examine the relative level of 84 genes related to EMT. Total RNA from 4 biological replicates of confluent HEK293 cells (control) and HEK293 cells exposed to IGROV1 cell-secreted exosomes (treatment) was isolated (see above). cDNA (1 ug) was made, diluted and qPCR was performed per manufacturer's instructions using the LightCycler 480 Real-Time PCR System (Roche Applied Science). The cycling parameters include an initial denaturation step of 10 minutes at 95°C followed by 45 cycles of repeating denaturing at 15 seconds at 95°C and annealing for 1 minute at 60°C with a final cooling step of 5 minutes at 37°C. Biosciences software associated with this Profiling Array was used to determine significant changes in expression level, and EMT-related genes with a fold change of at least 3 were reported.

### 2.7. Western Blot Analysis

Cells and exosomes were lysed in M-PER (Thermo Scientific, 78501) supplemented with Halt proteinase inhibitor cocktail (Thermo Scientific, 1 : 100, 87786) and phenylmethanesulfonyl fluoride solution (Boston BioProducts, 1 : 100, PI-120). Cells were centrifuged at 14,000 g for 5 minutes at 4°C, and protein concentration was determined using the bicinchoninic assay (BCA) method (Pierce BCA Protein Assay Kit, Thermo Scientific, 23225). 30 ug of protein from cell lysates and 40 ug of protein from exosomal lysates were diluted in 6x buffer/DTT loading dye and heated to 95°C for 10 minutes, as described previously [11, 33, 34]. Protein was electrophoresed to 4–20% Ready Gel Tris-HCl Precast Gels (Bio-Rad, 161-1159) at 90 V for 15 minutes followed by 120 V for 1 hour, and transfer onto a nitrocellulose membrane for 1 hour at 100 V on ice. The membrane was washed with 1X TBST for 5 min and blocked at room temperature for 1 hour with 5% dry milk in 1X TBST. The membrane was washed three times for 5 minutes in 1X TBST and incubated with the following primary antibodies: LIN28A (1 : 1000 rabbit polyclonal, ab63740, Abcam), LIN28B (1 : 1000 rabbit polyclonal, 4196S, Cell Signaling Technology), GAPDH (1 : 3000, rabbit polyclonal, ab37168), CYTO C (1 : 100, mouse monoclonal IgG_2b_, sc13156, Santa Cruz Biotechnology), TSG101 (1 : 500, rabbit polyclonal, 14497-1-AP, Proteintech), or EPCAM (1 : 500, rabbit polyclonal, 21050-1-AP, Proteintech). Primary antibodies were resuspended in 5% milk 1X TBST and membranes were incubated overnight at 4°C, except for GAPDH in which case the membranes were incubated 1 hour at room temperature. Membranes were washed three times for 5 minutes each in 1X TBST before the secondary antibodies were applied for 1 hour at room temperature. The secondary antibodies are as follows: goat anti-rabbit IgG-HRP (1 : 5000, sc-2004, Santa Cruz Biotechnology) was for LIN28A, LIN28B, and GAPDH, goat anti-mouse IgG-HRP (1 : 2000, sc-2031, Santa Cruz Biotechnology) was used with CYTO C, and goat anti-rabbit IgG-HRP (1 : 2000, ab6721, Abcam) was used with TSG101 and EPCAM. Membranes were washed with 1X TBST 3 times for 5 minutes and incubated for 5 minutes in ECL Prime Western Blotting Detection Reagent (Amersham, RPN2209) and for 1 second in SuperSignal West Dura Extended Duration Substrate (Thermo Scientific, 34075) for chemiluminescence detection on the ChemiDoc MP System. Image Lab 4.1 software was used to determine densitometry.

Experiments were carried out using at least two independent biological replicates and the experiments were repeated. Densitometry was calculated by dividing the band volume of the gene of interest over the housekeeping gene GAPDH. Statistical analysis was determined by ANOVA followed by Tukey pairwise comparison (Minitab 17). *p* values less than 0.05 were considered statistically significant.

### 2.8. Exosome Transfer

For exosome transfers, 1 × 10^6^ IGROV1-CD63-GFP cells were seeded onto 10 cm cell plates (Celltreat, 229690) in complete RPMI 1640 vesicle-depleted medium and grown for 3 days. IGROV1 and OV420 cell-secreted exosomes were isolated from the culture medium using the exosome isolation procedure described above and stained with Vybrant DiD cell-labeling solution (Invitrogen, V22887) per manufacturer's instructions. 24 hours before exosomes were isolated; 5 × 10^4^ HEK293 cells were grown in complete DMEM vesicle-depleted medium to approximately 60–80% confluency in 4-well plates. Exosomes were resuspended in 500 *μ*L of complete DMEM vesicle-depleted medium and were added onto 5 × 10^4^ HEK293 cells, referred to as exosome transfer, for 96 hours. The first control (vehicle) was 500 *μ*L of complete DMEM vesicle-depleted medium plated onto 5 × 10^4^ HEK293 cells as a negative control. The second control (supernatant) included 500 *μ*L of nonprecipitated, supernatant/ExoQuick-TC biofluid plated onto 5 × 10^4^ HEK293 cells. Cells were imaged on the Olympus FSX100 Bio Imaging Navigator using the FSX100 software or the LSM 510 Meta 405 Confocal Microscope System Zeiss. A series of images were collected at 1 *μ*m intervals and used to generate a Z-stack. After 96 hours, HEK293 cells were trypsinized and snap frozen in liquid nitrogen and stored in −80°C for RNA/protein analysis or used immediately for migration and invasion assays.

### 2.9. Migration and Invasion Assays

Migration and invasion assays were performed using the 24-well 8.0 *μ*m BD BioCoat Tumor Invasion System (BD Biosciences, 354166) and the BD Falcon 24-multiwell 8 *μ*m insert system (BD Biosciences, 351158) as per manufacturer's instructions. Briefly, cell monolayers were pretreated with 10 ug/mL DiIC_12_(3) in 10% FBS DMEM vesicle-depleted medium for 1 hour at 37°C. Cells were trypsinized in 0.1% FBS DMEM vesicle-depleted serum-free medium. 5 × 10^4^ cells were seeded onto the apical chambers. Chemoattractant of either 10% FBS DMEM vesicle-depleted medium (positive control) or 0.1% FBS DMEM vesicle-depleted serum-free medium (negative control) was added to the basal chambers. The BD BioCoat Tumor Invasion System and the uncoated BD Falcon FluoroBlok 24-Multiwell System were incubated for 48 hours at 37°C, 5% CO_2_. Readings were taken every 6 hours, for 48 hours, with Syngene HT and Gen5 program using 530/25-excitation filter and 590/35-emission filter, and sensitivity was set at 52. Fluorescence of invaded/migrated cells was read at wavelengths 549/565 nm (Ex/Em) for detection of DiI_12_C. Background fluorescence was subtracted and data reduction was performed by subtracting values of the negative from the positive control. Migration and invasion relative fluorescence units were plotted separately, and experiments were carried out at least two times with three independent biological replicates.

## 3. Results

### 3.1. LIN28A and LIN28B Expression in IGROV1, OV420, and HEK293 Cells

**Figure 1:**
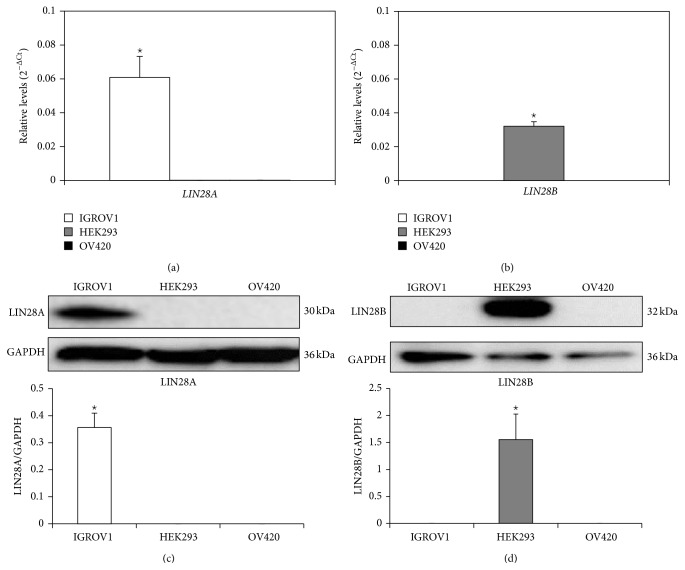
Levels of LIN28A and LIN28B mRNA and protein in IGROV1, HEK293, and OV420 cells. qPCR was performed to obtain* LIN28A* (a) and* LIN28B* (b) mRNA levels. Data were normalized against the geometric mean of* GAPDH*,* MRPS15*, and* TBP*. Western blot analysis was performed to obtain LIN28A (c) and LIN28B (d) protein levels. Densitometry of bands was determined to calculate relative protein amount. Asterisk indicates a *p* value <0.05.

qPCR analysis revealed significantly higher levels of* LIN28A* in IGROV1 compared to OV420 or HEK293 cells (Figure 1(a)). Alternatively, HEK293 cells were the only cells with detectable amounts of* LIN28B* (Figure 1(b)), whereas OV420 cells did not contain any detectable amounts of* LIN28A* or* LIN28B*. These results were confirmed for LIN28A and LIN28B protein by Western blot analysis (Figures 1(c) and 1(d)).

### 3.2. let-7 miRNA Level in IGROV1, OV420, and HEK293 Cells

**Figure 2:**
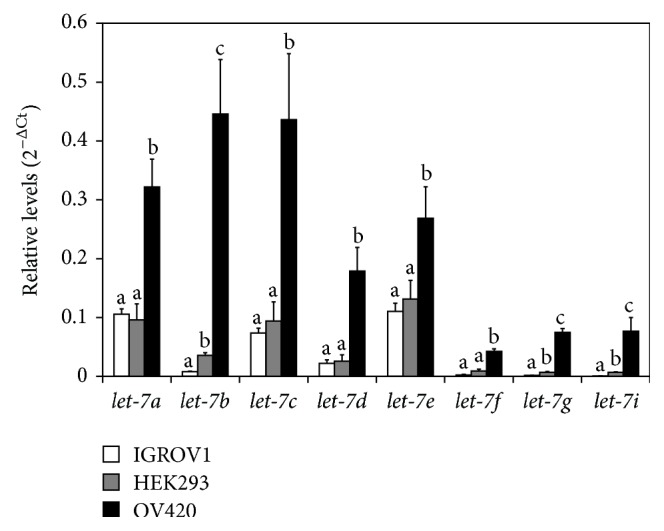
Relative level of* let-7* miRNAs in IGROV1, OV420, and HEK293 cells. qPCR data were normalized against the U6 snRNA. Means without the same superscript are significantly different (*p* value <0.05).

LIN28A and LIN28B are known regulators of* let-7* miRNAs by inhibiting their maturation. qPCR analysis revealed that the relative levels of mature* let-7a*,* let-7b*,* let-7c*,* let-7d*,* let-7e*,* let-7f*,* let-7g*, and* let-7i* are all significantly higher in OV420 cells compared to both IGROV1 and HEK293 cells (Figure 2).

### 3.3. Uptake of IGROV1 and OV420 Secreted Exosomes by HEK293 Cells

**Figure 3:**
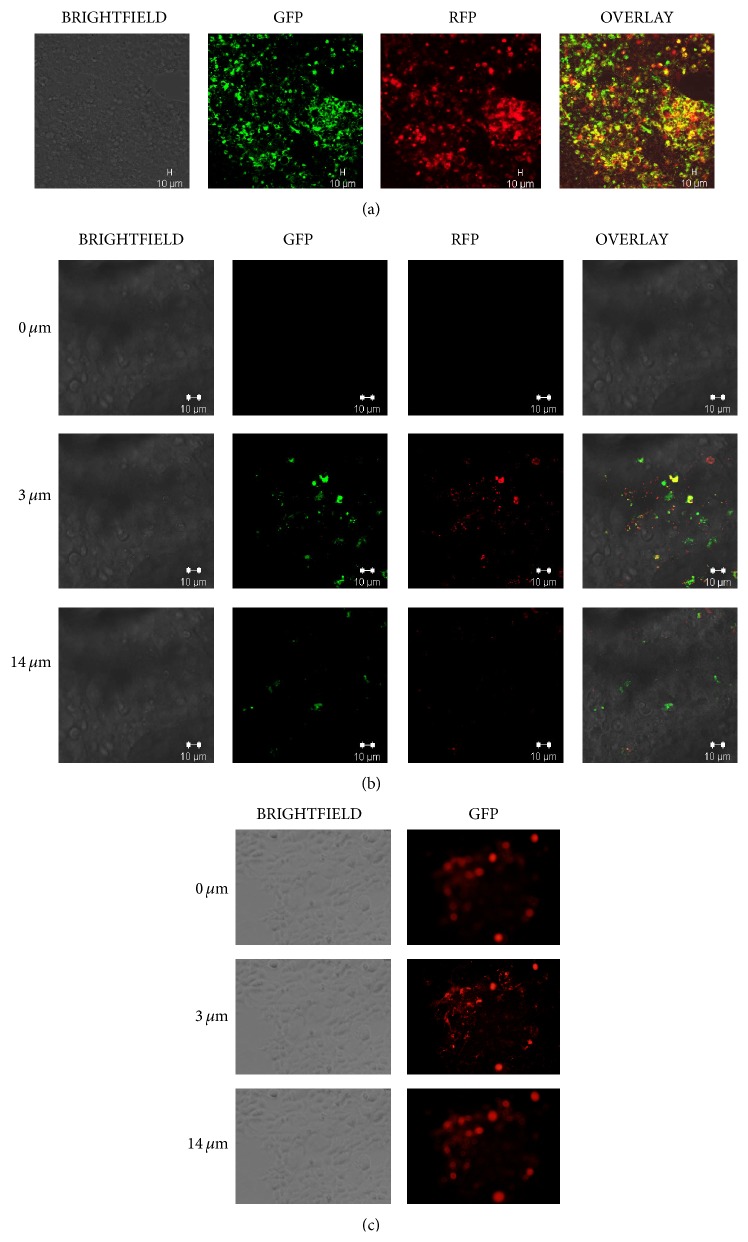
Detection of exosome-uptake by HEK293 cells. (a) HEK293 cells after exposure to CD63-GFP positive (green) and DiD-RFP labeled (red) IGROV1 secreted exosomes. 20x magnification was utilized to image HEK293 cells following exosome exposure using confocal microscopy. (b) Z-stack image of HEK293 cells after exposure to CD63-GFP positive (green) and DiD-RFP labeled (red) IGROV1 secreted exosomes. (c) Z-stack image of HEK293 cells after exposure to OV420 secreted, DiD labeled (red) exosomes. 40x magnification was utilized to image HEK293 cells following exosome exposure using confocal microscopy.

IGROV1 cells were infected with CD63-GFP-cytotracer, which led to GFP-labeling of exosomes by the host cells. In addition, exosomes secreted by OV420 cells were incubated with the DiD cell-labeling solution, which labels lipids in cell membranes. HEK293 cell incubation with GFP-labeled IGROV1 secreted exosomes or DiD-labeled OV420 secreted exosomes leads to uptake of these exosomes as evident by Z-stack imaging (Figure 3).

### 3.4. HEK293 Cell Invasion and Migration following Uptake of IGROV1 and OV420 Cell-Secreted Exosomes

**Figure 4:**
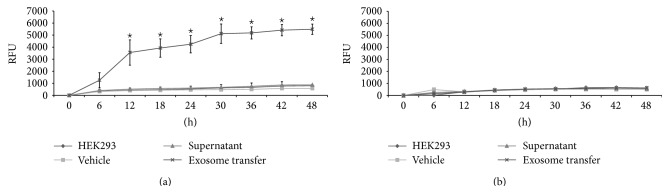
Invasion of HEK293 cells exposed to IGROV1 or OV420 cell-secreted exosomes. (a) HEK293 cells invasion when exposed to IGROV1 cell-secreted exosomes and (b) HEK293 cells invasion when exposed to OV420 cell-secreted exosomes. Asterisk indicates a *p* value <0.05.

**Figure 5:**
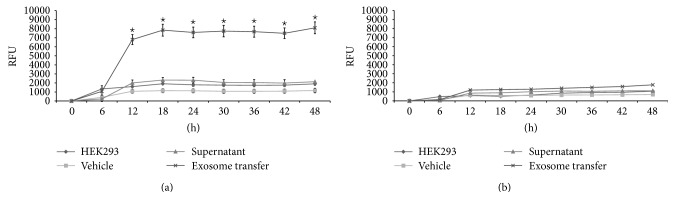
Migration of HEK293 cells exposed to IGROV1 or OV420 cell-secreted exosomes. (a) HEK293 cells migration when exposed to IGROV1 cell-secreted exosomes and (b) HEK293 cells migration when exposed to OV420 cell-secreted exosomes. Asterisk indicates a *p* value <0.05.

To determine if uptake of IGROV1 or OV420 cell-secreted exosomes leads to changes in cell phenotype or behavior, HEK293 cells were incubated with exosomes and invasion and migration assays were conducted. Uptake of IGROV1 cell-secreted exosomes leads to significant increase in invasion as well as migration as early as 12 hours in HEK293 cells, compared to HEK293 cells incubated in media with vehicle or supernatant (Figures 4 and 5). Uptake of OV420 cell-secreted exosomes had no effect on HEK293 cell invasion or migration. These results mimic what is seen for IGROV1 and OV420 cells, in that IGROV1 cells are significantly more invasive than OV420 cells (Supplemental Figure).

### 3.5. Relative Levels of Epithelial-to-Mesenchymal Transition (EMT) Related Genes in HEK293 Cells following Uptake of IGROV1 Cell-Secreted Exosomes

HEK293 exposure to and uptake by IGROV1 cell-secreted exosomes leads to invasion and migration; therefore, qPCR analysis was performed using Human Epithelial-to-Mesenchymal Transition RT^2^ Profiler PCR arrays to determine changes in relative expression of 84 genes known to be involved in EMT. Exposure to exosomes resulted in significant increased levels of 45 EMT-related genes, including* ZEB1*,* NOTCH1*,* WNT5A*,* NODAL*, and* SNAI2* (Table 2).

### 3.6. LIN28 and miRNAs in HEK293 Cells following Uptake of IGROV1 Cell-Secreted Exosomes

**Figure 6:**
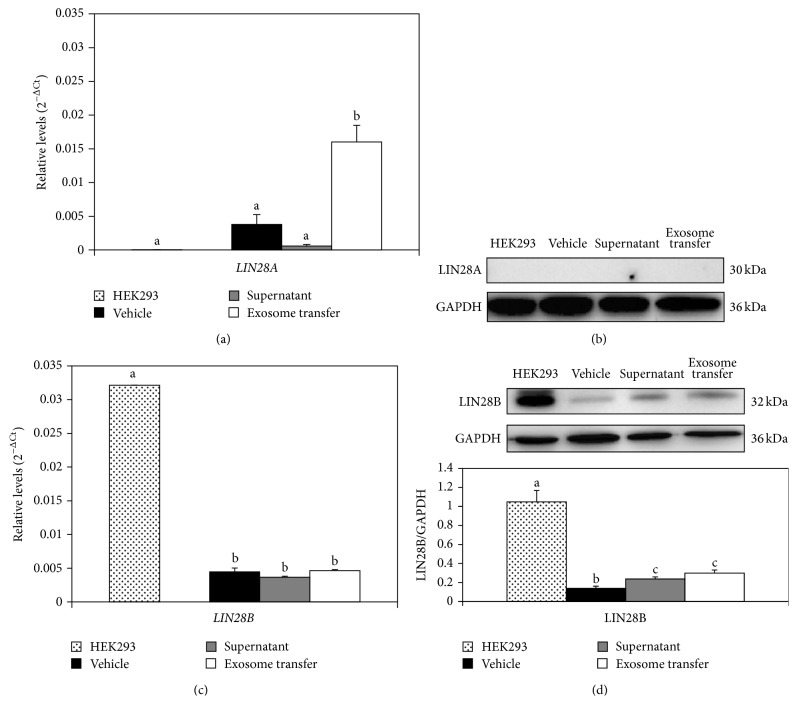
Relative levels of LIN28A and LIN28B mRNA and protein in HEK293 cells exposed to IGROV1 cell-secreted exosomes. HEK293 cells treated with vesicle-deplete IGROV1 conditioned media (vehicle), HEK293 cells treated with supernatant from exosome pellet (supernatant), and HEK293 cells treated with exosomes (exosome transfer). qPCR was performed to assess mRNA levels of* LIN28A* (a) and* LIN28B* (c) after HEK293 cells were exposed to IGROV1 cell-secreted exosomes. Data were normalized against the geometric mean of* GAPDH*,* MRPS15*, and* TBP*. Means without the same superscript are significantly different (*p* value <0.05). Western blot analysis was performed to determine LIN28A (b) and LIN28B (d) protein levels after HEK293 cells were exposed to IGROV1 cell-secreted exosomes. Densitometry of bands was determined to calculate relative protein amount. Means without the same superscript are significantly different (*p* value <0.05).

**Figure 7:**
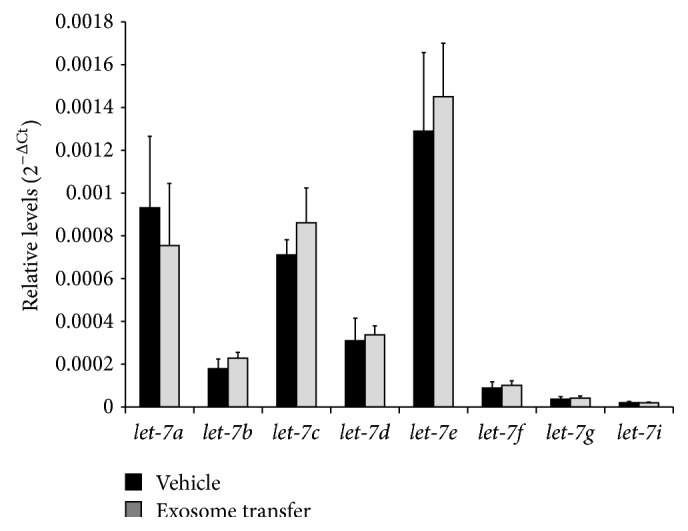
Relative level of* let-7* miRNAs in HEK293 cells following IGROV1 cell-secreted exosome treatment. qPCR was utilized to determine the relative levels and data were normalized against U6 snRNA.

**Figure 8:**
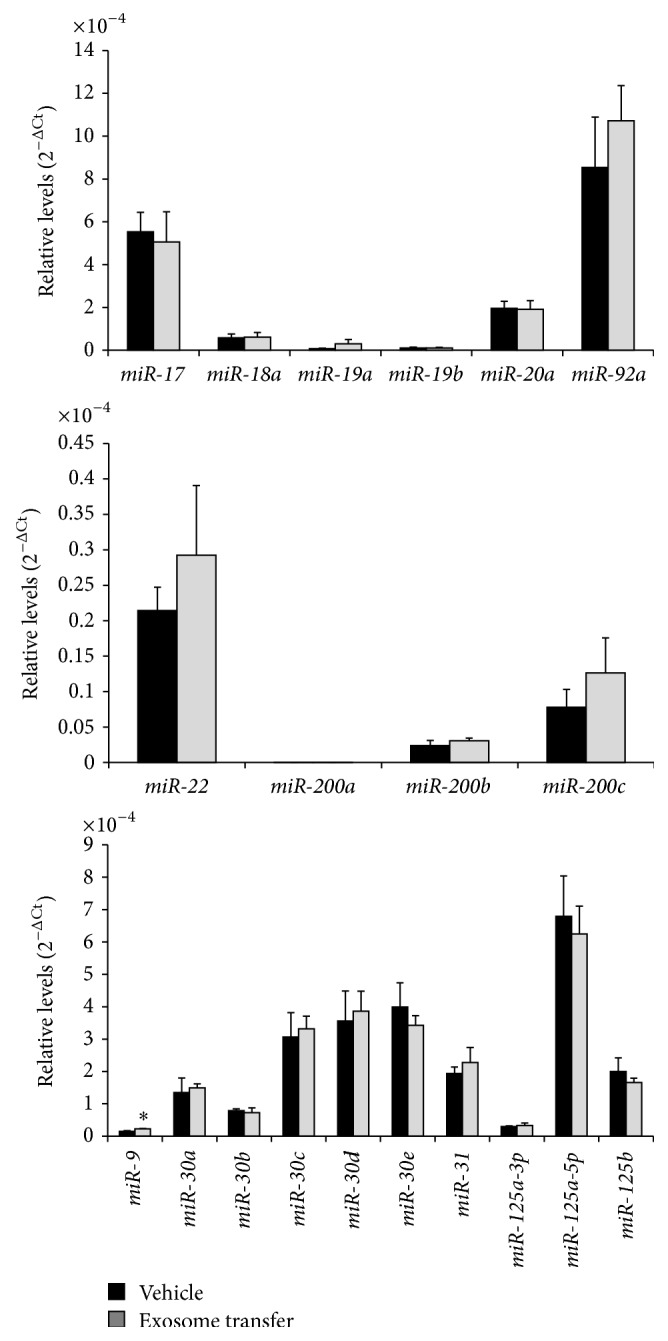
Relative level of selected miRNAs in HEK293 cells following IGROV1 cell-secreted exosome treatment. qPCR was utilized to determine the relative levels and data were normalized against U6 snRNA. Asterisks indicate a *p* value <0.05.

In addition to EMT-related genes, relative changes in LIN28A, LIN28B, and selected miRNAs known to be involved in EMT and cancer (oncomirs) were assessed in HEK293 cells following uptake of IGROV1 cell-secreted exosomes. Taqman qPCR assays revealed that there was a significant, 15-fold increase in* LIN28A* level in HEK293 cells exposed to IGROV1 cell-secreted exosomes compared to IGROV1 cell conditioned media depleted of exosomes (Figure 6(a)), but not* LIN28B* (Figure 6(c)). This change was significant although no change in LIN28A (Figure 6(b)) or LIN28B (Figure 6(d)) protein level was evident in HEK293 cell following exposure to IGROV1 cell-secreted exosomes. No change in* let-7* (*a, b, c, d, ef, g*, and* i*) miRNA was observed in HEK293 cells following treatment with IGROV1 cell-secreted exosomes (Figure 7). Moreover, qPCR analysis of selected other EMT and cancer associated miRNAs, that is,* miR-17, miR-18a, miR-19a, miR-19b, miR-20a, miR-92, miR-22, miR-200a, miR-9, miR-30b, miR-30d, miR-30e, miR-125a-3p, miR-125-5p*, and* miR-125b*, indicates a small but significant increase in* miR-9* alone in HEK293 cells following treatment with IGROV1 cell-secreted exosomes (Figure 8).

### 3.7. LIN28 and miRNAs in IGROV1 and OV420 Cell-Secreted Exosomes

**Figure 9:**
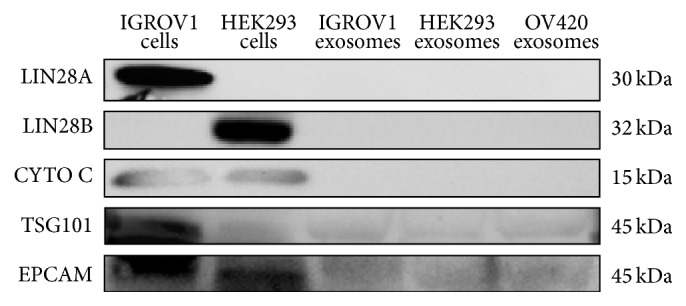
Detection of LIN28A and LIN28B protein in exosomes. Western blot was used to determine the presence of LIN28A protein in exosomes (columns 3–5). The cytoplasmic/mitochondrial protein cytochrome C (CYTO C) was used as a negative control to exclude nonexosomal fractions, and tumor susceptibility gene 101 (TSG101), a component of the endosomal sorting, and EPCAM (epithelial cell adhesion molecule) are used as positive controls for exosomes.

**Figure 10:**
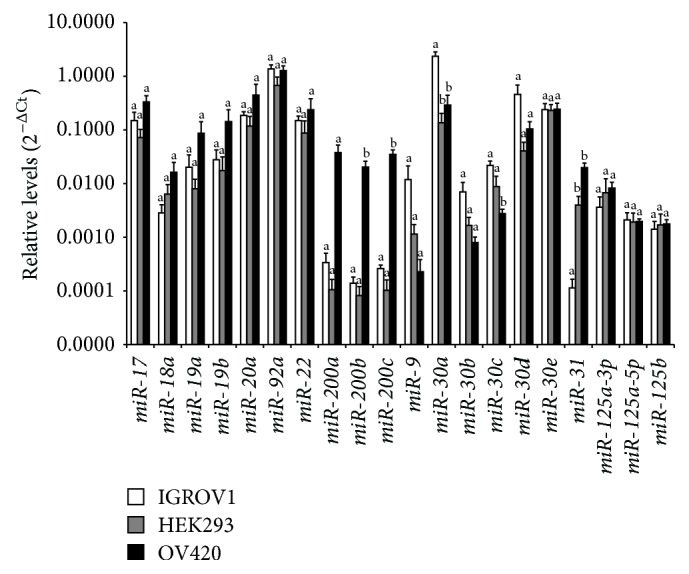
Relative levels of selected miRNAs in ovarian cancer cell-secreted exosomes. qPCR data were normalized against U6 snRNA. Means without the same superscript are significantly different (*p* value <0.05).

**Figure 11:**
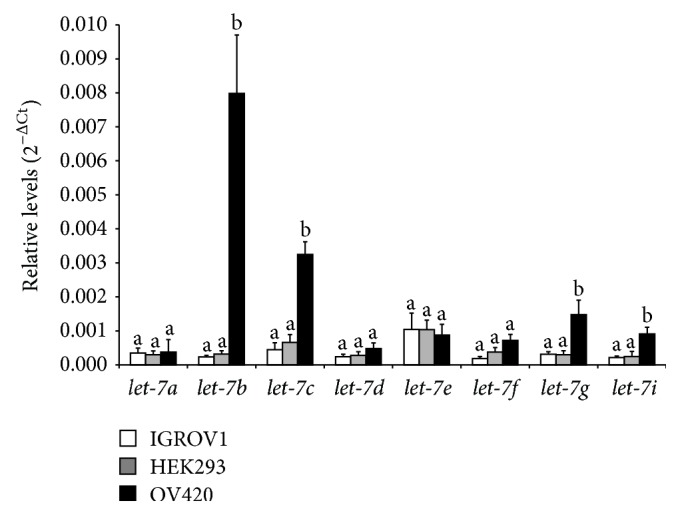
Relative levels of* let-7* miRNAs in ovarian cancer cell-secreted exosomes. qPCR data were normalized against U6 snRNA. Means without the same superscript are significantly different (*p* value <0.05).

Using Western blot analysis, exosomes secreted by IGROV1, OV420, and HEK293 cells were all positive for exosomal protein markers TSG101 and EPCAM; however, neither LIN28A nor LIN28B was detected in IGROV1, OV420, or HEK293 cell-secreted exosomes (Figure 9). qPCR analysis of miRNAs previously associated with cancer (*miR-17, miR-18a, miR-19a, miR-19b, miR-20a, miR-92, miR-22, miR-200a, miR-9, miR-30b, miR-30d, miR-30e, miR-125a-3p, miR-125-5p*, and* miR-125b*) in exosomes secreted by IGROV1, OV420, and HEK293 cells indicates that overall miRNA levels were similar with the exception of* miR-200b* and* miR-200c*, which were significantly elevated in OV420 cells,* miR30a*, which was significantly higher in IGROV1 exosomes,* miR-30c*, which was significantly higher in IGROV1 and HEK293 exosomes, and* miR-31*, which was significantly higher in OV420 and HEK293 exosomes (Figure 10). Finally,* let-7*b,* let-7*c,* let-7*g, and* let-7*i miRNAs were significantly higher in OV420 compared to IGROV1 and HEK293 secreted exosomes (Figure 11).

## 4. Discussion

In this study we sought to determine if exosomes from high LIN28A expressing ovarian cancer cells could be taken up by HEK293 cells leading to changes in gene expression and cell phenotype. IGROV1 cells contain high levels of LIN28A mRNA and protein and low levels of* let-7* miRNAs, confirming a previous report [35]. Conversely, OV420 ovarian cancer cells contain no detectable amount of LIN28A or LIN28B, and* let-7* miRNAs are significantly higher in these cells compared to IGROV1 cells. HEK293 cells were selected as target cells for IGROV1 and OV420 cell-secreted exosomes, as these cells are readily used and not cancer derived. Interestingly, HEK293 cells were found to contain LIN28B, although this amount was less compared to relative LIN28A levels in IGROV1 cells. Currently it is unclear what the different functions of LIN28A and LIN28B are; both regulate* let-7* miRNA maturation with LIN28A functioning in the cytoplasm, whereas LIN28B appears to sequester* let-7s* to the nucleus [35].

Previous studies demonstrate that tumor secreted microvesicles/exosomes can alter target cell gene expression and cell behavior. For example, glioblastoma tumor cell-secreted exosomes in the brain are enriched with angiogenic proteins and can be taken up by brain microvascular endothelial cells to stimulate tubal formation [4]. More recently, Le and colleagues [36] report that metastatic mouse and human breast cancer cells secrete extracellular vesicles (including exosomes), containing* miR-200s*,* miR-200s*, and can be transferred to nonmetastatic cells. Furthermore, breast cancer cell-secreted exosomes containing* miR-122* induced tumor colonization in brain and lung in xenograft mouse model [37]. Our data reveals that exosomes secreted by high LIN28A expressing IGROV1 cells, but not low LIN28 expressing OV420 cells, alter nonovarian HEK293 cell behavior and lead to an increase in invasion and migration. An invasion assay was conducted on IGROV1 and OV420 cells. After 48 hrs IGROV1 cells exhibited significantly higher invasion compared to OV420 cells and support data that exosomes secreted by IGROV1 but not OV420 cells are able to induce invasion in HEK293 cells (Figure S1). Moreover, relative expression levels of several genes related to EMT were upregulated following exposure to IGROV1 cell-secreted exosomes, which could explain the altered behavior of HEK293 cells.

LIN28 is a RNA-binding protein that binds to and regulates both mRNAs and miRNAs. LIN28A and miRNAs can reprogram cells and are known regulators of cell differentiation, and studies have demonstrated deregulation of miRNAs in cancer [38–41]. However, LIN28A protein was not detected in IGROV1 cell-secreted exosomes, and no change in LIN28A was observed in HEK293 cells following exposure to IGROV1 exosomes. Interestingly, we did observe that endogenous LIN28B levels were lower in HEK293 cells when cultured in vesicle-depleted media compared to complete media. The reason for this is unclear at this point.

We also determined that* let-7*b,* let-7*c,* let-7*g, and* let-7*i are significantly lower in IGROV1 exosomes compared to OV420 exosomes, which reflect what was found in the donor cells. Profiling of miRNAs in epithelial ovarian tissues demonstrated* let-7*s were lower in these tissues [42], and lower levels of* let-7*i are found in patients resistant to chemotherapy as well as patients with a poor prognosis in late-stage ovarian cancer [43]. A recent study described exosomal* miR-200* and* let-7* families from SKOV3 cells (highly invasive) and OVCAR-3 cells (low invasiveness), and it was determined that* miR-200* and* let-7* families are sequestered in exosomes of more invasive ovarian cancer cells lines [44]. However, Taylor and Gercel-Taylor [5] report that the expression pattern of* let-7* family is similar between donor cells and exosomes, similar to our findings.

The* miR-17-92, miR-200*, and* miR-30* families are all known regulators of oncogenesis and EMT [45–48]. Therefore, we examined the presence of these miRNAs in exosomes, as well as HEK293 cells following exposure to IGROV1 cell-secreted exosomes. Interestingly, although* miR-200b, miR-200c*, and* miR-31* are lower and* miR-30a* and* miR-30c* are higher in IGROV1 cell-secreted exosomes compared to OV420 exosomes, relative levels of these miRNAs were not different in HEK293 cells following exposure to IGROV1 cell-secreted exosomes. Our results did reveal that* miR-9* was significantly higher in HEK293 cells after IGROV1 exosome exposure.* miR-9* regulates snail family zinc finger 2 (*SNAI2*) [49], also known as* SLUG*, a necessary factor in type II tumor formation.* SNAI2* was significantly higher in HEK293 cells following exposure to IGROV1 cell-secreted exosomes. It is unclear whether exosomal* miR-9* actually was transferred from IGROV1 to HEK293 cells and whether it directly regulates* SNAI2* transcription and/or translation.

Taken together, data presented here demonstrate that high LIN28A expressing ovarian cancer cells secrete exosomes that, when taken up by nonmetastatic target cells, induce EMT-related gene expression and invasion and migration.

## 5. Conclusions

Our results demonstrate that the more metastatic, high LIN28A expressing IGROV1 cells secrete exosomes that can upregulate genes related to EMT and induce invasion and migration in HEK293 cells. We were unable to demonstrate presence in or transfer of LIN28 itself by exosomes, and it is possible that the observed changes in cell phenotype and gene expression induced by IGROV1 cell-secreted exosomes are not due to LIN28 directly. Future studies using different high LIN28A expressing cancer cells will be important to determine if the observed features of IGROV1 cell-secreted exosomes hold true for other high LIN28A-expressing cells. Although a number of known cancer and EMT-related miRNAs were investigated, only miR-9 was altered in HEK293 cells following IGROV1 cell-secreted exosome treatment. The fact that LIN28 can interact with and bind to many RNAs in addition to miRNAs suggests additional RNAs are potential candidates that can be loaded into exosomes and/or transferred to target cells. Future RNAseq experiments will provide insight into the underlying mechanism of the observed exosome-induced invasion and migration.

## Supplementary Material

An invasion assay was conducted on IGROV1 and OV420 cells. After 48 hrs IGROV1 cells exhibited significantly higher invasion compared to OV420 cells and support data that exosomes secreted by IGROV1 but not OV420 cells are able to induce invasion in HEK293 cells.

## Figures and Tables

**Table 1:** Human miRNA sequences used for qPCR experiments. These sequences were obtained from miRBase program.

miRNA	Sequence (5′-3′)
*hsa-let-7a *	UGAGGUAGUAGGUUGUAUAGUU
*hsa-let-7b *	UGAGGUAGUAGGUUGUGUGGUU
*hsa-let-7c *	UGAGGUAGUAGGUUGUAUGGUU
*hsa-let-7d *	AGAGGUAGUAGGUUGCAUAGUU
*hsa-let-7e *	UGAGGUAGGAGGUUGUAUAGUU
*hsa-let-7f *	UGAGGUAGUAGAUUGUAUAGUU
*hsa-let-7g *	UGAGGUAGUAGUUUGUACAGUU
*hsa-let-7i *	UGAGGUAGUAGUUUGUGCUGUU
*hsa-let-7a-3p *	CUAUACAAUCUACUGUCUUUC
*hsa-let-7-f1-3p *	CUAUACAAUCUAUUGCCUUCCC
*hsa-miR-9 *	UCUUUGGUUAUCUAGCUGUAUGA
*hsa-miR-17 *	CAAAGUGCUUACAGUGCAGGUAG
*hsa-miR-18a *	UAAGGUGCAUCUAGUGCAGAUAG
*hsa-miR-19a *	UGUGCAAAUCUAUGCAAAACUGA
*hsa-miR-19b *	UGUGCAAAUCCAUGCAAAACUGA
*hsa-miR-20a *	UAAAGUGCUUAUAGUGCAGGUAG
*hsa-miR-22 *	AAGCUGCCAGUUGAAGAACUGU
*hsa-miR-30a *	UGUAAACAUCCUCGACUGGAAG
*hsa-miR-30b *	UGUAAACAUCCUACACUCAGCU
*hsa-miR-30c *	UGUAAACAUCCUACACUCUCAGC
*hsa-miR-30d *	UGUAAACAUCCCCGACUGGAAG
*hsa-miR-30e *	UGUAAACAUCCUUGACUGGAAG
*hsa-miR-31 *	AGGCAAGAUGCUGGCAUAGCU
*hsa-miR-92a *	UAUUGCACUUGUCCCGGCCUGU
*hsa-miR-200a *	UAACACUGUCUGGUAACGAUGU
*hsa-miR-200b *	UAAUACUGCCUGGUAAUGAUGA
*hsa-miR-200c *	UAAUACUGCCGGGUAAUGAUGGA
*hsa-miR-125a-3p *	ACAGGUGAGGUUCUUGGGAGCC
*hsa-miR-125a-5p *	UCCCUGAGACCCUUUAACCUGUGA
*hsa-miR-125b *	UCCCUGAGACCCUAACUUGUGA
*Htman t6 snRNA (U6) *	CGCAAGGAUGACACGCAAAUUC

**Table 2:** Epithelial-to-Mesenchymal Transition (EMT) related genes that are significantly upregulated after HEK293 cells exposed to IGROV1 cell-secreted exosomes.

Fold change	Genes
25.65	*TIMP1 *
10.95	*FOXC2 *
10.55	*NOTCH1 *
9.67	*PTP4A1 *
8.32	*GNG11 *
7.37	*SNAI1 *
7.33	*FGFBP1 *
7.13	*SOX10 *
6.58	*COL3A1 *
6.49	*JAG1 *
6.45	*WNT5A *
6.41	*SNAI3 *
6.38	*NODAL *
6.35	*CDH1 *
6.32	*IL1RN *
6.04	*TMEM132A *
5.7	*COL1A2 *
5.53	*GSC *
5.39	*MMP3 *
5.35	*ERBB3 *
5.32	*SNAI2 *
5.24	*BMP1 *
5.2	*KRT7 *
5.15	*MST1R *
5.13	*PTK2 *
4.82	*F11R *
4.78	*AHNAK *
4.49	*IGFBP4 *
4.29	*FZD7 *
4.28	*TWIST1 *
4.19	*ITGA5 *
4.14	*WNT5B *
4.12	*STAT3 *
4.09	*BMP7 *
4	*MMP9 *
3.95	*SPP1 *
3.86	*SPARC *
3.82	*SERPINE1 *
3.81	*ILK *
3.8	*PLEK2 *
3.37	*WNT11 *
3.25	*TMEFF1 *
3.22	*TGFB1 *
3.09	*TGFB3 *
3.03	*ZEB1 *
