# Supplementary figures and images for: High LIN28A Expressing Ovarian Cancer Cells Secrete Exosomes That Induce Invasion and Migration in HEK293 Cells
Source: Biomed Res Int. 2015 Oct 25;2015:701390. doi: 10.1155/2015/701390 (PMC4637063; doi:10.1155/2015/701390)

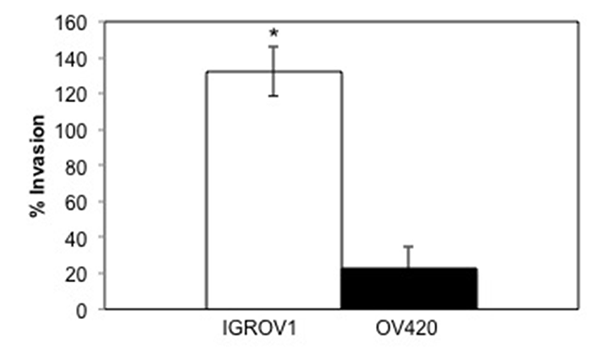

Supplement: Supplementary file 1 — An invasion assay was conducted on IGROV1 and OV420 cells. After 48 hrs IGROV1 cells exhibited significantly higher invasion compared to OV420 cells and support data that exosomes secreted by IGROV1 but not OV420 cells are able to induce invasion in HEK293 cells. [file 701390.f1.tif]
